# MicroRNA-424/503 cluster members regulate bovine granulosa cell proliferation and cell cycle progression by targeting SMAD7 gene through activin signalling pathway

**DOI:** 10.1186/s13048-018-0410-3

**Published:** 2018-05-01

**Authors:** Hari Om Pande, Dawit Tesfaye, Michael Hoelker, Samuel Gebremedhn, Eva Held, Christiane Neuhoff, Ernst Tholen, Karl Schellander, Dessie Salilew Wondim

**Affiliations:** 1 Department of Animal Breeding and Husbandry, Institute of Animal Science, University of Bonn, Endenicher Allee 15, 53115 Bonn, Germany; 2 Teaching and Research Station Frankenforst, Faculty of Agriculture, University of Bonn, Königswinter, Germany; 3 Center of Integrated Dairy Research, University of Bonn, Bonn, Germany

**Keywords:** miR-424/503 cluster members, Granulosa cells, SMAD7, ACVR2A, Activin a

## Abstract

**Background:**

The granulosa cells are indispensable for follicular development and its function is orchestrated by several genes, which in turn posttranscriptionally regulated by microRNAs (miRNA). In our previous study, the miRRNA-424/503 cluster was found to be highly abundant in bovine granulosa cells (bGCs) of preovulatory dominant follicle compared to subordinate counterpart at day 19 of the bovine estrous cycle. Other study also indicated the involvement of miR-424/503 cluster in tumour cell resistance to apoptosis suggesting this miRNA cluster may involve in cell survival. However, the role of miR-424/503 cluster in granulosa cell function remains elusive Therefore, this study aimed to investigate the role of miRNA-424/503 cluster in bGCs function using microRNA gain- and loss-of-function approaches.

**Results:**

The role of miR-424/503 cluster members in granulosa cell function was investigated by overexpressing or inhibiting its activity in vitro cultured granulosa cells using miR-424/503 mimic or inhibitor, respectively. Luciferase reporter assay showed that *SMAD7* and *ACVR2A* are the direct targets of the miRNA-424/503 cluster members. In line with this, overexpression of miRNA-424/503 cluster members using its mimic and inhibition of its activity by its inhibitor reduced and increased, respectively the expression of *SMAD7* and *ACVR2A*. Furthermore, flow cytometric analysis indicated that overexpression of miRNA-424/503 cluster members enhanced bGCs proliferation by promoting G1- to S- phase cell cycle transition. Modulation of miRNA-424/503 cluster members tended to increase phosphorylation of SMAD2/3 in the Activin signalling pathway. Moreover, sequence specific knockdown of *SMAD7,* the target gene of miRNA-424/503 cluster members, using small interfering RNA also revealed similar phenotypic and molecular alterations observed when miRNA-424/503 cluster members were overexpressed. Similarly, to get more insight about the role of miRNA-424/503 cluster members in activin signalling pathway, granulosa cells were treated with activin A. Activin A treatment increased cell proliferation and downregulation of both miRNA-424/503 members and its target gene, indicated the presence of negative feedback loop between activin A and the expression of miRNA-424/503.

**Conclusion:**

This study suggests that the miRNA-424/503 cluster members are involved in regulating bovine granulosa cell proliferation and cell cycle progression. Further, miRNA-424/503 cluster members target the *SMAD7* and *ACVR2A* genes which are involved in the activin signalling pathway.

**Electronic supplementary material:**

The online version of this article (10.1186/s13048-018-0410-3) contains supplementary material, which is available to authorized users.

## Background

The mammalian follicle, consisting of an oocyte surrounded by granulosa and theca cells, represents the basic functional unit of the ovary [1]. The growth of the obligatory gonadotropin-dependent follicle, which is a complex but nevertheless well-coordinated process, occurs in a wave-like fashion, with two to three waves per oestrous cycle [2] followed by ovulation or atresia. Among the follicular cells, granulosa cells are critically indispensable for the growth and maturation of follicles, and they undergo a series of morphological and functional changes [3]. The primordial follicle, holding an arrested oocyte at diplotene stage, is enclosed by a single flattened layer of somatic granulosa cells (GCs) [4]. Further, the sequential, well-controlled transformation from the primordial to antral follicle stage is the result of the differentiation and proliferation of GCs that provide essential and vital inputs in the form of steroid hormones, cytokines, and paracrine and autocrine factors during the process of follicular development [5–7], which is tightly regulated by array of genes [3, 5, 6, 8, 9] that may in turn fine-tuned by microRNAs [10, 11].

To better understand the genetic regulation of granulosa cell function and their role in follicular development, several transcriptome profiling studies have been conducted to examine the expression patterns of genes in bovine granulosa cells (bGCs) [8] at different phases of antral follicle growth [9]. Accordingly, several genes involved in steroidogenesis (*CYP17A1*, *CYP11A1*, *HSD3B1*, *STAR*), cell proliferation/the cell cycle (*CCND2*, *PCNA*), gonadotropin receptors (*LHCGR*, *FSHR*) and growth factors (*GDF9*, *BMP2*, Activins, *IGF1*, *IGF2*) have been found to be altered in granulosa cells depending on the size and stage of follicular development [3, 12–16].

Granulosa cell proliferation and differentiation have been reported as important cellular activity within dominant follicle in the late phase of estrous cycle [17, 18]. STAR is a key molecule in steroidogenesis and is a marker of granulosa cell differentiation [19].

These molecular cues, which are responsible for follicular development, are under the control of several epigenetic mechanisms, including microRNAs (miRNAs). These small noncoding RNAs, ~ 20–22 nucleotides in length, are epigenetic regulators that control gene expression post-transcriptionally by targeting the 3´-UTR in a sequence-specific manner leading to mRNA degradation or translation inhibition [10, 11].

MicroRNAs play crucial roles in almost all biological functions, including cell proliferation, differentiation, and apoptosis [12, 13], and disturb function of miRNAs are associated with various diseases such as cancer [20]. Since the discovery of first miRNA (Lin-4) in 1993 [10, 21], a continuing major challenge has been deciphering the functional aspects of miRNAs in bio-physiology, including mammalian reproduction. Previous evidences support the involvement of miRNAs in follicular growth and development through the regulation of granulosa cell proliferation, differentiation, apoptosis and steroidogenesis [22–28]. However, limited numbers of studies have validated the role of miRNAs in bovine follicular development [12, 13, 23].

Previously, we have reported the expression pattern of miRNAs in bovine granulosa cells of subordinate and dominant follicles during the early luteal phase (day 3 and day 7) [29] and late follicular phase (day 19) [23] of the bovine oestrous cycle and their possible association with follicular recruitment, selection and dominance. In the latter study, of the 64 total differentially expressed miRNAs, the miR-424/503 cluster was significantly enriched in the granulosa cells of preovulatory dominant follicles. Inline to this, recent studies have demonstrated that the miR-424/503 cluster enhances tumour cell resistance to apoptosis [30], coordinates the remodelling of the epithelium in the involution of mammary gland [31], and reverses chemo-resistance via T-cell immune response activation by blocking the PD-L1 immune checkpoint [32]. Nonetheless, the involvement of the miR-424/503 cluster in reproductive functions remains elusive. With in-silico analysis mothers against decapentaplegic homolog 7 (*SMAD7*) and activin receptor type 2A (*ACVR2A*) were identified as target genes of miR-424/503, which are associated with the activin signalling pathway of the TGF-β superfamily members and a known key regulator of follicle development in mammals [33]. In the present study, we demonstrated that miR-424/503 cluster epigenetically regulates bovine granulosa cell function by targeting SMAD7, and through fine tuning of the activin signalling pathway.

## Methods

### MicroRNA target gene prediction

The mature sequences of miR-424-5p (miR-424) and miR-503-5p (miR-503) were obtained from the miRBase (http://www.mirbase.org/) database. We performed an in silico target prediction for potential putative targets using the miRWalk database (http://zmf.umm.uni-heidelberg.de/apps/zmf/mirwalk/). The miRNA-mRNA binding site prediction in bovine sequences was performed using TargetScan 6.2 [34]. Target gene predictions were considered according to rank based on the predicted efficacy or targeting as calculated using ‘cumulative weighted context++ scores’ of the sites [34] and probability of conserved targeting (P_CT_) [35]. Accordingly, among several genes, the *SMAD7* [36] and *ACVR2A* genes, which are ubiquitously expressed in the ovarian follicle and important in reproductive performance [37], were selected for functional analysis. The secondary structure of miR-424 and miR-503 was predicted by RNAhybrid (http://bibiserv.techfak.uni-bielefeld.de/rnahybrid).

### Bovine granulosa cell culture and transfection

Bovine ovaries as sources of bGCs were collected from a local slaughterhouse. Ovaries were processed to obtain follicular fluid and isolation of granulosa cells as described previously [12]. Further, a total of 2.0–2.5 × 10^5^ bGCs per well were seeded into CytoOne® 24-well plate (Starlab International GmbH, Germany) in the F12+ culture media. The bGCs were cultured in 37 °C with 5% CO_2_ in humidified environment. The bGCs were incubated for 48 h to attach and pre-confluent (60–70%) for treatment or transfection purpose. In the culture medium FSH, IGF1 or other factors were not added to avoid its effect on bovine granulosa cell proliferation. In some experiments cells were cultured in the presence of Recombinant Human/Mouse/Rat Activin A (R&D Systems, MN, USA).

The chemically synthesized miRNA-424-5p mimic and inhibitor, miR-503-5p mimic and inhibitor, and the corresponding negative controls (NC) were used to transfect (Qiagen GmbH, Germany) bGCs. The miRNAs and/or plasmids were diluted in Opti-MEM I reduced-serum medium (Invitrogen). Sub-confluent cultured bGCs (70–80% confluent) were co-transfected with 500 ng of the wild-type or mutant-construct plasmid and 50 nM individual microRNA mimic or mimic control. For miR-424/503 gain- and loss-of-function analysis, 50 nM individual microRNA mimic, inhibitor or corresponding negative controls were co-transfected to sub-confluent cultured bGCs. The transfection was performed using Lipofectamine 2000 transfection reagent (Life Technologies, Germany).

### Plasmid construction and luciferase assay

**Fig. 1:**
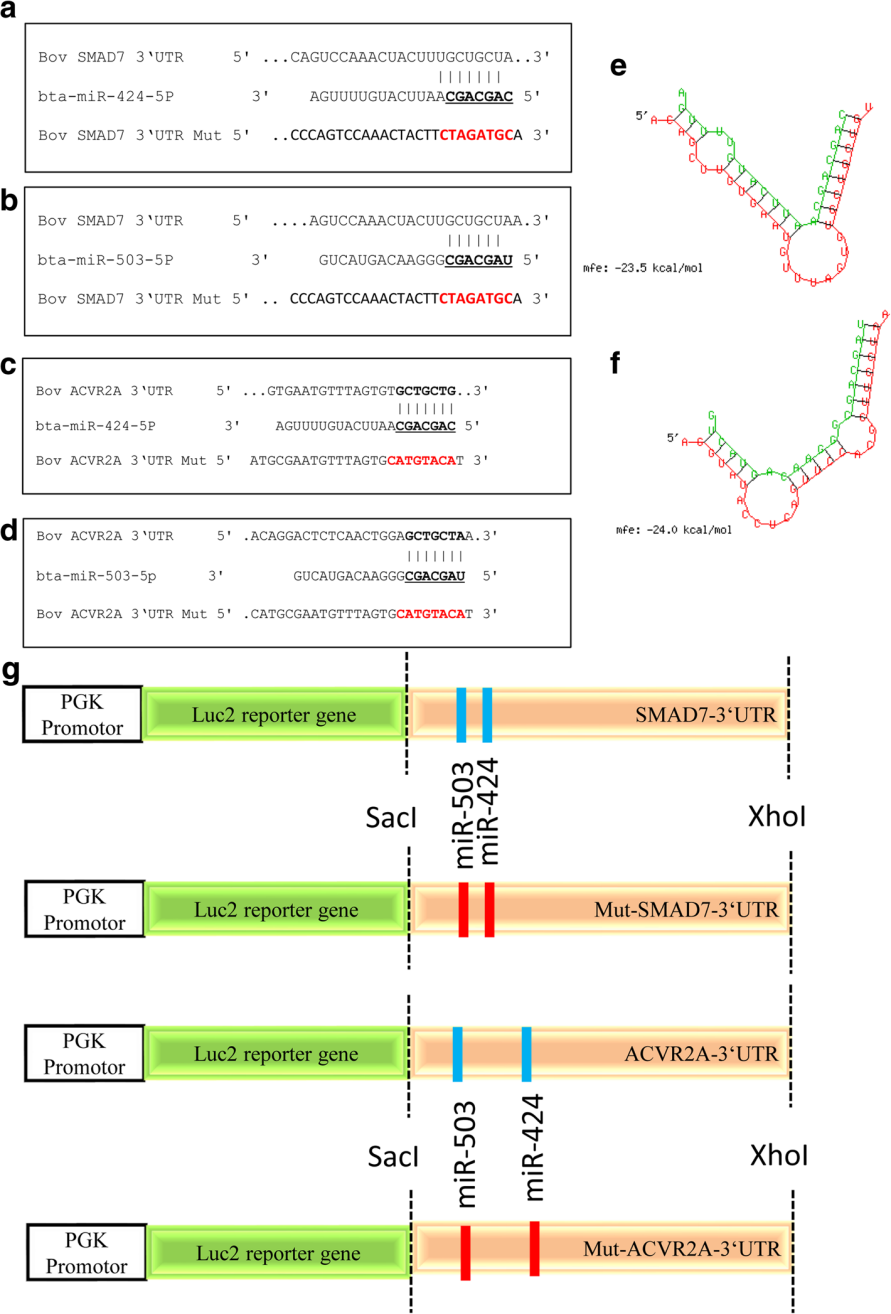
The miRNA-mRNA binding sites in bovine SMAD7 3´-UTR (**a**, **b**) and ACVR2A 3´-UTR sequences (**c**, **d**), Bold and underlined letters indicate putative binding sites and mutated regions. The minimum free energies (kcal/mol) of miR-424 (**e**) and miR-503 (**f**). Schematic diagram of the reporter constructs containing the putative miRNA-mRNA binding sites of the bovine SMAD7 and ACVR2A 3´-UTR sequences (**g**)

To validate whether the *SMAD7* and *ACVR2A* gene are real targets of the miR-424/503 cluster, fragments of the 3´-UTR of SMAD7 or 3´-UTR of ACVR2A containing the binding sites for miR-424-5p (miR-424) and miR-503-5p (miR-503) (wild type) or with mutations in the seed sequences of miR-424/503 (mutant type) (Fig. 1) were cloned and inserted between the *Sac*I and *Xho*I restriction sites of the pmirGLO Dual-Luciferase miRNA Target Expression Vector (Promega Corporation, USA). The cDNA from ovarian bGCs was used to amplify the predicted miRNA-mRNA binding site in the 3´-UTR region of the *SMAD7* or *ACVR2A* mRNA. Specific primers and 50-mer mutated oligonucleotides were designed based on bovine *SMAD7* (XM_005224232.3) or *ACVR2A* (NM_174227) mRNA sequences in GenBank (Additional file 1: Table S1). The luciferase activity was measured 48 h after transfection using the pmirGLO Dual Luciferase® Reporter Assay System (Promega Corporation, USA) according to the manufacturer’s protocol. Firefly and *Renilla* luciferase activity was detected by measuring the absorbance on a Centro LB 960 Microplate Luminometer (Berthold Technologies GmbH, Germany).

### Total RNA isolation and cDNA synthesis

To confirm the expression of target and marker genes in treated bGCs at each stage of the experiment, harvested bGCs were resuspended in lysis buffer, and the subsequent RNA isolation was performed using an miRNeasy® mini kit (Qiagen GmbH, Germany) following the manufacturer’s protocol. Total RNA quantity and purity (260/280 ratios) was measured with a NanoDrop 8000 spectrophotometer (NanoDrop products, USA). After validating the quality and concentration of the RNA samples, the cDNA synthesis was performed using a RevertAid first stand cDNA synthesis kit (Thermo Fisher Scientific, Germany). Briefly, each RNA sample (1 μg) was co-incubated with 1 μl of Oligo (dT)_18_ primer and dH_2_O to a total of 11 μl at 65 °C for 5 min and then chilled on ice for 5 min. The reverse transcription was performed in a total mixture volume of 20 μl consisting of 4 μl of 5× Reaction Buffer, 1 μl of RevertAid Reverse Transcriptase, 2 μl of dNTP mix and 1 μl of RiboLock RNase Inhibitor. The reactions were carried out in a thermocycler programmed at 37 °C for 60 min followed by 70 °C for 5 min.

### Quantitative real-time polymerase chain reaction (qRT-PCR)

Primers for specific genes were designed using the Primer-BLAST program (http://www.ncbi.nlm.nih.gov/tools/primer-blast/). The details of the primers are described in Additional file 1: Table S2. The specificity of each primer amplicon was confirmed by sequencing the PCR products using a GenomeLab GeXP Genetic Analysis System (Beckman Coulter GmbH, Germany). The qRT-PCR analysis of mRNA was performed in an Applied Biosystem® StepOnePlus™ System (Thermo Fisher Scientific Inc., USA), using iTaq™ Universal SYBR® Green Supermix (Bio-Rad Laboratories GmbH, Germany), with the program described previously [23, 29] The mRNA expression data were analysed using the comparative Ct (2^-∆∆Ct^) method [38], and β-ACTIN was used as an internal control.

Candidate miRNAs were quantified as described previously [23, 29]. Briefly, cDNA was synthesized using 80 ng of miRNA-enriched total RNA using a miRCURY LNA™ Universal cDNA synthesis kit (Exiqon, Denmark) according to the manufacturer’s instructions. The synthesized cDNA was diluted 40× and used for the qRT-PCR analysis of candidate miRNAs using ExiLENT SYBR Green Master mix (Exiqon, Denmark). The thermal cycling program was used as described previously [23, 29]. The specificity of the miRNA amplification was evaluated by melting curve analysis. The 5 s ribosomal RNA (5 s rRNA) (miRCURY LNA™ Universal RT microRNA PCR) was used as the reference gene primer. The qRT-PCR data were analysed using the comparative Ct (2^-∆∆Ct^) method [38].

### Western blot analysis

Total protein from cultured bGCs was isolated using 1× PLB (Promega Corporation, USA). The total protein concentration was determined using the Bradford method [39]. Western blotting was performed as described previously [12, 13]. The antibodies used were (Santa Cruz Biotechnology Inc., Germany): anti-ACTR-II2A goat polyclonal antibody (product no. sc-5667), anti-SMAD7 rabbit polyclonal antibody (product no. sc-11,392), anti-PCNA rabbit polyclonal antibody (product no. sc-7907), anti-STAR rabbit polyclonal antibody (product no. sc-25,806), anti-SMAD2/3 rabbit poly clonal antibody (product no.sct-5678), anti-psmad2/3 rabbit monoclonal antibody (sct-8828) or anti-β-ACTIN mouse monoclonal antibody (product no. sc-47,778). At the end of the incubation period, the membrane was washed six times with 1× TBST and incubated with the corresponding donkey anti-goat, goat anti-rabbit, or goat anti-mouse secondary antibody conjugated to horseradish peroxidase (Santa Cruz Biotechnology). The detection of the protein signal was then performed using Clarity Western ECL Substrate (Bio-Rad Laboratories). The relative band intensity was determined by ImageJ program (https://imagej.nih.gov/ij/).

### Cell proliferation assay

A total of 2 × 10^4^ bGCs per well were seeded into a 96-well plate as described previously [12, 13] Individual miR-424/503 mimics, inhibitors or corresponding controls were transfected into sub-confluent cultured bGCs (70–80% confluent). After 48 h of incubation, 10 μl of CCK-8 kit solution (Dojindo EU GmbH, Germany) was added to each well, and the plate was incubated for another 2 h. The optical density (OD) at a wavelength of 450 nm was measured using a Synergy™ H1 Multi-Mode Reader (BioTek Instruments Inc., Germany).

### Cell cycle assay

Cultured granulosa cells were transfected with 75 nM miR-424/503 cluster miRNA mimics, inhibitors, or the corresponding negative control (NC) and SMAD7 siRNA, ACVR2A siRNA or NC siRNA. The cells were trypsinized 48 h later and collected in a 15-mL Falcon tube (Thermo Fisher Scientific, Germany), followed by centrifugation at 750×*g* for 5 min and washing twice with 1 x CMF-PBS. A minimum of ~ 1 × 10^6^ cells were fixed in ice-cold 70% ethanol at 4 °C overnight. The cells were then centrifuged briefly, and the cell pellet was washed twice with 500 μl of 1× CMF-PBS. The cells were then labelled with 50 μg/mL propidium iodide (PI) and treated with 50 μg/mL RNase. The cells were then incubated at 37 °C for 30 min and processed in a BD LSRFortessa™ flow cytometer (BD Biosciences). The cell cycle distribution was analysed using ModFit LT software (http://www.vsh.com/products/mflt/index.asp).

### Targeted suppression of the SMAD7 and ACVR2A gene using small interfering RNA (siRNA)

Bovine specific LNA™ longRNA GapmeRs (Exiqon, USA) were used to inhibit the expression of SMAD7 (5´-TTCGCAGAGTCGGCTA-3′ and 5´-CGATTTTGCTCCGTA-3′) ACVR2A (5´-GTTACTGGATTCGACG-3′ and 5´-GTTGGTCAGTAATCTA-3′). The transfection of 75 nM SMAD7 siRNA, ACVR2A siRNA or control siRNA was performed as described above. The gene expression analysis and cell proliferation assays were performed as described above.

### Data analysis

All the quantitative data are presented as the mean ± standard error (SEM) and at least three biological replicates were used for each analysis. The statistical analysis was performed using GraphPad Prism® 5, version 5.02. The statistical significance between the mean values of two treatment groups was determined using a two-tailed Student’s t-test. However, data from more than two treatment groups were analysed using a one-way ANOVA followed by Dunnett’s post hoc test. The *p*-values indicate the statistical significance as described in each figure legend.

## Results

### SMAD7 and ACVR2A are the direct targets of the miRNA-424/503 cluster members

**Fig. 2:**
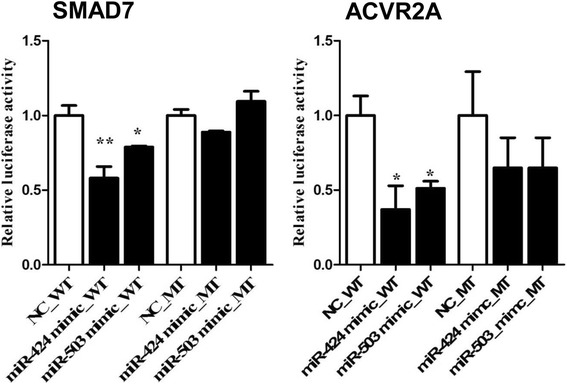
SMAD7 and ACVR2A are the direct targets of the miR-424/503 cluster members. Relative luciferase activity in bovine granulosa cells co-transfected with pmiRGLO vector harbouring the wild-type (WT) or mutant (MT) 3´-UTR sequence of SMAD7 or ACVR2A and the corresponding miRNA mimics or negative controls (NC) for miR-424 and miR-503. The data are presented as the mean ± SEM (*p < 0.05, **p < 0.01)

We demonstrated in our previous study that miR-424/503 cluster miRNAs were upregulated in the granulosa cells of preovulatory dominant follicles at day 19 of the oestrous cycle. MicroRNA-424/503 cluster members are transcribed from an intergenic region of chromosome X, which is polycistronic in nature and evolutionary conserved in mammals. The precursors of bta-miR-424-5p (miR-424) and bta-miR-503-5p (miR-503) are 96 bp and 83 bp, respectively. The mature sequences of bta-424-5p and miR-503-5p are CAGCAGCAAUUCAUGUUUUGA and UAGCAGCGGGAACAGUACUG, respectively, which are conserved in other mammalian species. Since miRNAs regulate biological functions by targeting the 3´-UTR of genes post-transcriptionally in a sequence-specific manner, we performed a computational prediction using TargetScan (http://www.targetscan.org) to generate an algorithm to identify putative targets, which revealed that miR-424 and miR-503 target *SMAD7* and *ACVR2A*. On the other side, miRNA prediction for SMAD7 target gene using TargetScan revealed that miR-424-5p was top predict with 8mer site type (an exact match to positions 2–8 of the mature miRNA (the seed + position 8) followed by an ‘A’); while, miR-503-5p showed 7mer-A1 site type (an exact match to positions 2–7 of the mature miRNA (the seed) followed by an ‘A’). The validation of the putative target genes, SMAD7 and ACVR2A, of the miR-424/503 cluster was performed by measuring the luciferase activity of an expression vector carrying the 3´-UTR of the *SMAD7* and *ACVR2A* gene, which contains the miRNA binding sites. The luciferase activity in bovine granulosa cells co-transfected with miR-424 and miR-503 mimics and a plasmid vector harbouring the wild-type SMAD7 and ACVR2A 3’-UTR was significantly reduced compared to bGCs transfected with a control for the individual miRNA mimics and the plasmid vector with the wild-type SMAD7 and ACVR2A 3´-UTR (*P* < 0.05; Fig. 2). However, there was no significant reduction in luciferase activity in bGCs co-transfected with individual miRNA mimics or a control and a plasmid vector harbouring a mutated SMAD7 and ACVR2A 3´-UTR sequence.

### Modulation of the miRNA-424/503 cluster members resulted in differential expression of SMAD7 and ACVR2A mRNA levels in cultured bovine granulosa cells

**Fig. 3:**
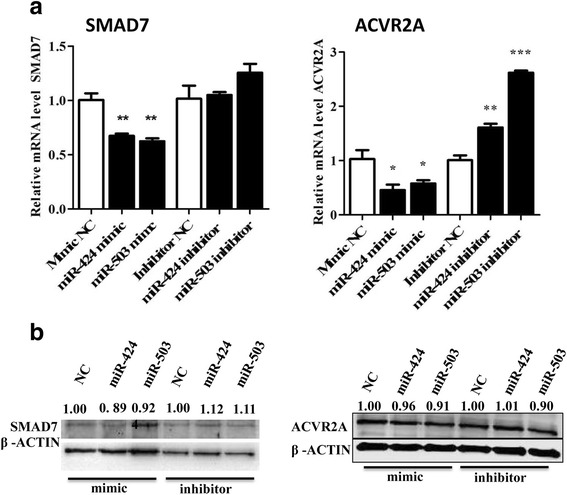
Modulation of the miR-424/503 cluster members altered the expression of SMAD7 and ACVR2A in bovine granulosa cells. Overexpression of miR-424 and miR-503 decreased the SMAD7 and ACVR2A mRNA (**a**) and protein (**b**) expression. The bar graphs indicate the mean ± SEM (*p < 0.05, **p < 0.01, ***p < 0.001). Numbers above western blots represent relative protein density

To obtain further insights into the role of the miR-424/503 cluster in granulosa cell function by regulating the expression of *SMAD7* and *ACVR2A* gene, we transfected cultured bGCs with either the miR-424/503 mimics, inhibitors or the corresponding controls at a concentration of 75 nM. The expression level of the *SMAD7* and *ACVR2A* mRNA was determined by qRT-PCR analysis 48 h after transfection. Transfection of the miR-424/503 cluster members mimic resulted in a significant reduction in the relative abundance of *SMAD7* (*P* < 0.01) and *ACVR2A* (*P* < 0.05) mRNA compared to the controls (Fig. 3a). Transfection of miR-424/503 inhibitors did not affect, the expression of *SMAD7* mRNA while the expression of *ACVR2A* mRNA was increased. The western blot analysis showed that bGCs transfected with miR-424/503 cluster mimics had no significant reduction in SMAD7 and ACVR2A protein levels compared to those transfected with negative controls The miR-424/503 cluster inhibitors had no significant change in SMAD7 and ACVR2A protein expression compared to those transfected with controls (Fig. 3b).

### Overexpression of the miRNA-424/503 cluster members enhanced the proliferation and reduced the differentiation of bovine granulosa cells

**Fig. 4:**
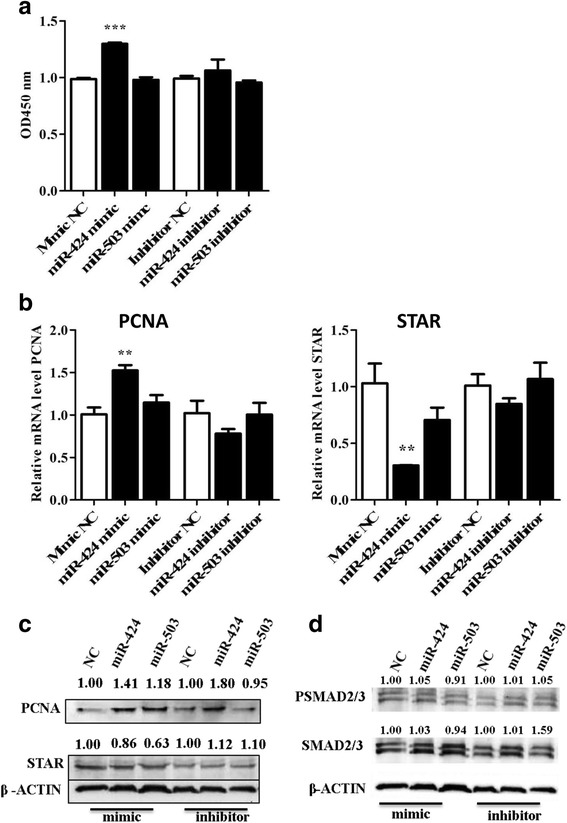
Overexpression of the miR-424/503 cluster members increased bovine granulosa cell proliferation. Cell proliferation assay of granulosa cells transfected with miR-424/503 cluster mimics and inhibitors and the corresponding negative control (NC) (**a**). The mRNA (**b**) and protein (**c**) expression level of PCNA and STAR in granulosa cells transfected with miR-424/503 cluster mimics, inhibitors or corresponding NC. The protein expression level of SMAD2/3 and phosphorylated SMAD2/3 in granulosa cells transfected with miR-424/503 cluster mimics and inhibitors and the corresponding NC (**d**). The bar graphs indicate the mean ± SEM (**p < 0.01, ***p < 0.001). Numbers above western blots represent relative protein density

MiR-424 mimic transfection in cultured bGCs significantly increased the proliferation activity in cells transfected with a miRNA mimic negative control (Fig. 4a). However, miR-503 had no significant effect on cell proliferation. Conversely, compared to the miRNA inhibitor control, bGCs transfected with miR-424 and miR-503 inhibitors showed no significant differences in proliferation activity. Moreover, the cell proliferation phenotype was accompanied by the higher expression level of the proliferation marker gene *PCNA* in the bGCs transfected with the miR-424 mimic compared to those transfected with the mimic control (*P* < 0.01). However, transfection of inhibitors resulted in a slight reduction in the expression of *PCNA* (Fig. 4b). The PCNA protein expression showed an increasing tendency in bovine granulosa cells transfected with the miR-424/503 cluster mimic (Fig. 4c).

As a marker of differentiation, the expression of the *STAR* gene was investigated after modulation of the miRNA cluster. Transfection of miR-424/503 cluster mimic resulted in a significant reduction in *STAR* gene expression compared to the negative control (Fig. 4b, c). These results suggest that miR-424 promotes bovine granulosa cell proliferation and decreases the terminal differentiation of the bGCs, which leads to follicular survival and development.

Furthermore, we investigated the role of miR-424/503 on downstream members of the activin signalling pathway; the western blot results showed that the expression of the SMAD2/3 proteins tended to increase after the overexpression of the miR-424 mimic compared to the negative control (4D). Moreover, the overexpression of the miR-424 mimic did not change the phosphorylated SMAD2/3 protein level (4D). Overexpression of miR-503 mimic could not increase the expression of SMAD2/3 or phosphorylated SMAD2/3 protein level compared to mimic negative control (4D).

### Overexpression of the miRNA-424/503 cluster members enhanced the cell cycle transition from G1- to S-phase in bovine granulosa cells

**Fig. 5:**
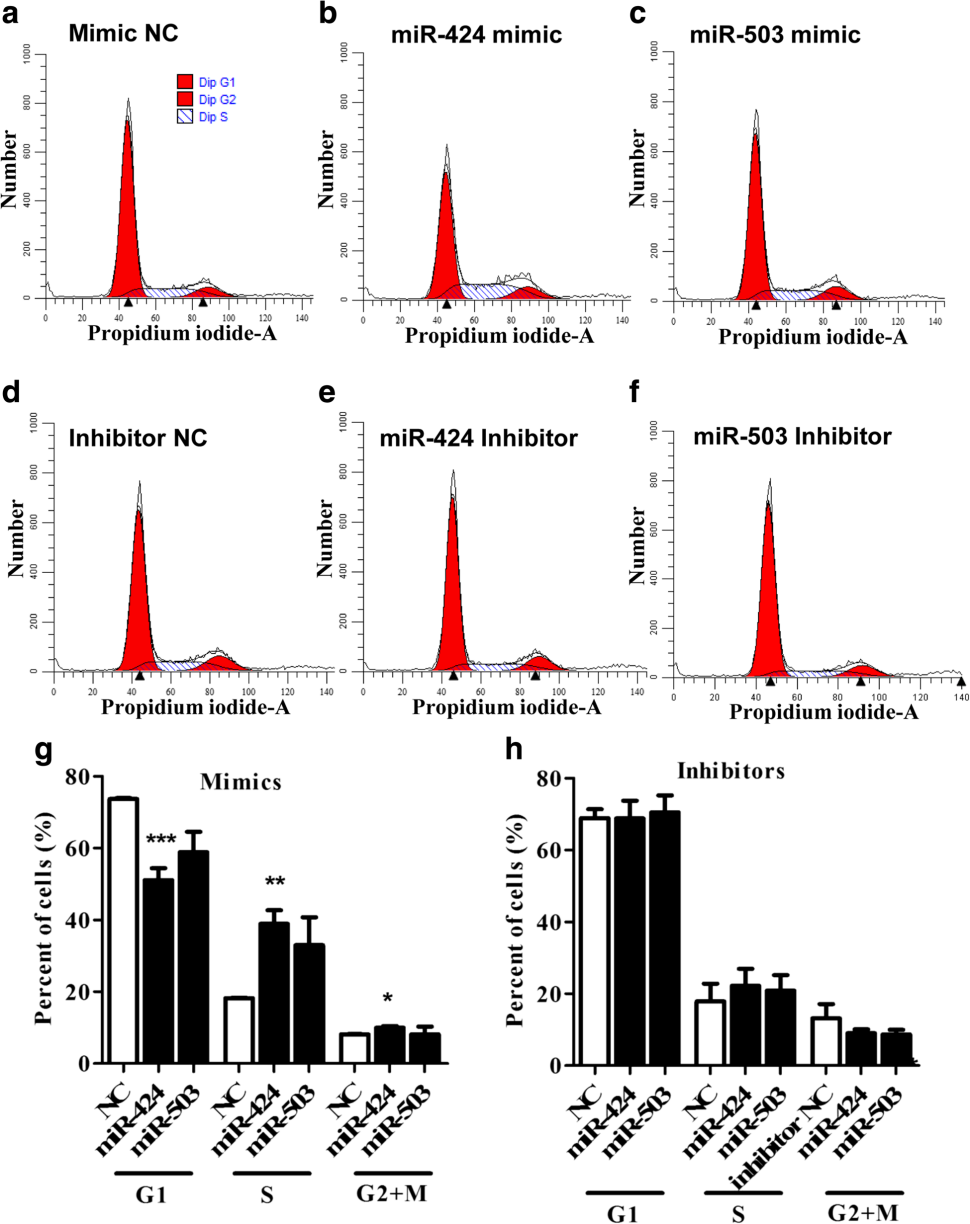
Overexpression of the miR-424/503 cluster members enhanced cell cycle progression of bovine granulosa cells. Flow cytometric analysis showing the cell cycle distribution (G1/G0, S and G2/M phases) of propidium iodide (PI)-labeled bovine granulosa cells transfected with the mimic negative control (NC) (**a**), miR-424 mimic (**b**), miR-503 mimic (**c**), inhibitor NC (**d**), miR-424 inhibitor (**e**), or miR-503 inhibitor (**f**). Bar graphs showing the percentages of cells in G1/G0, S and G2/M phases of the cell cycle after transfection with miR-424/503 cluster mimics (**g**), and inhibitors (**h**). The bar graphs indicate the mean ± SEM (*p < 0.05, **p < 0.01, ***p < 0.001)

To further confirm the proliferation results and to understand the role of the miR-424/503 cluster in modulating the cell cycle of bGCs, we performed flow cytometric analysis after transfection of the miR-424/503 cluster members. The overexpression of the miR-424 mimic resulted in a significant reduction (*P* < 0.001) in the number of cells in G0/G1-phase, while a significant increase (*P* < 0.01) in the percentage of cells in S-phase was observed compared to cells transfected with the mimic negative control (Fig. 5g). This result indicated that the overexpression of miR-424 could increase bovine granulosa cell proliferation by promoting G1- to S-phase cell cycle transition. In contrast, the miR-424/503 cluster inhibitor did not cause any measurable changes in the cell cycle profile of bovine granulosa cells (Fig. 5h).

### Effect of sequence specific knockdown of the *SMAD7* and *ACVR2A* gene in bovine granulosa cells

**Fig. 6:**
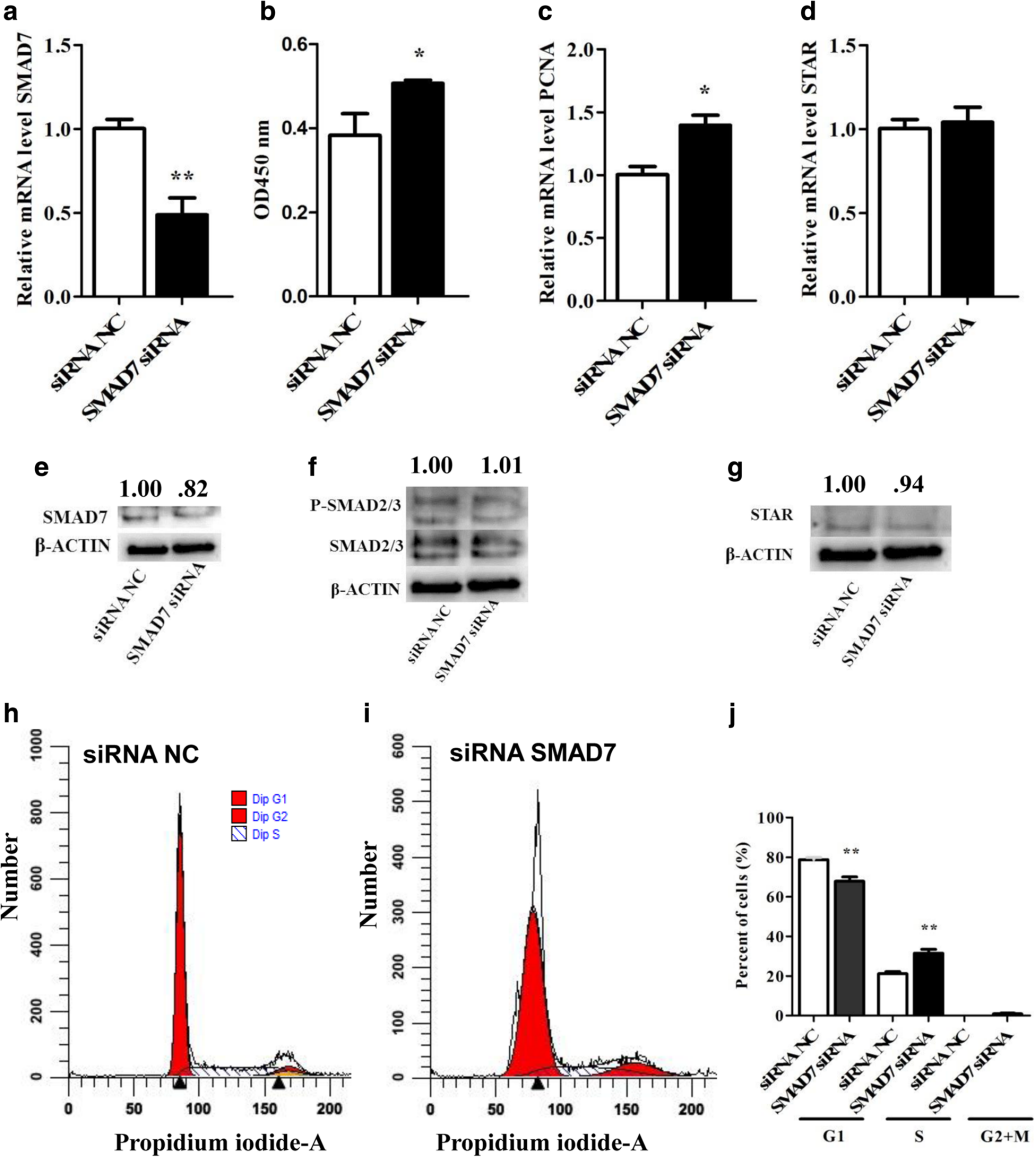
Sequence-specific knockdown of SMAD7 increased granulosa cell proliferation. The mRNA (**a**) and protein expression level (**e**) of SMAD7, mRNA level of PCNA (**c**), mRNA (**d**) and protein expression level (**g**) of STAR in granulosa cells transfected with SMAD7 siRNA or siRNA negative control (NC). The proliferation activity in granulosa cells transfected with SMAD7 siRNA (**b**). The protein expression level of SMAD2/3 and PSMAD2/3 in granulosa cells transfected with SMAD7 siRNA or siRNA negative control (NC) (**f**). Flow cytometric analysis showing the cell cycle distribution (G1/G0, S and G2/M phases) of propidium iodide (PI)-labeled bovine granulosa cells transfected with the SMAD7 siRNA (**i**) or siRNA negative control (NC) (**h**). Bar graphs showing the percentages of cells in G1/G0, S and G2/M phases of the cell cycle in cells transfected SMAD7 siRNA or siRNA negative control (NC) (**j**). The bar graphs indicate the mean ± SEM (**p < 0.01). Numbers above western blots represent relative protein density

In order to investigate the involvement of SMAD7 in bGCs proliferation and differentiation, and to further validate the regulatory role of the miR-424/503 cluster in *SMAD7* expression, we performed an independent experiment by knocking down the expression of *SMAD7* using small interfering RNA (siRNA). Bovine GCs transfected with the SMAD7 siRNA showed a significant reduction in the expression of both *SMAD7* mRNA (*P* < 0.05) and protein compared to cells transfected with the siRNA negative control (Fig. 6a, e). The transfection of bGCs with the SMAD7 siRNA significantly enhanced proliferation of bGCs compared to siRNA negative control (Fig. 6b), which was accompanied by noteworthy change in the expression of the marker of proliferation *PCNA* (Fig. 6c). However, flow cytometric analysis showed reduction (*P* < 0.001) in the number of cells in G0/G1-phase (Fig. 6j), while a significant increase (*P* < 0.01) in the percentage of cells in S-phase (Fig. 6j) was observed compared to cells transfected following knocking down of *SMAD7* with siRNA compared to negative control. Further, the suppression of the *SMAD7* gene using siRNA had no effect on the expression of the *STAR* gene and STAR protein (Fig. 6d, g). Western blot analysis showed that the expression of SMAD2/3 and phosphorylated SMAD2/3 was slightly increased in siRNA-transfected cells compared to cells transfected with the negative control (Fig. 6f).

**Fig. 7:**
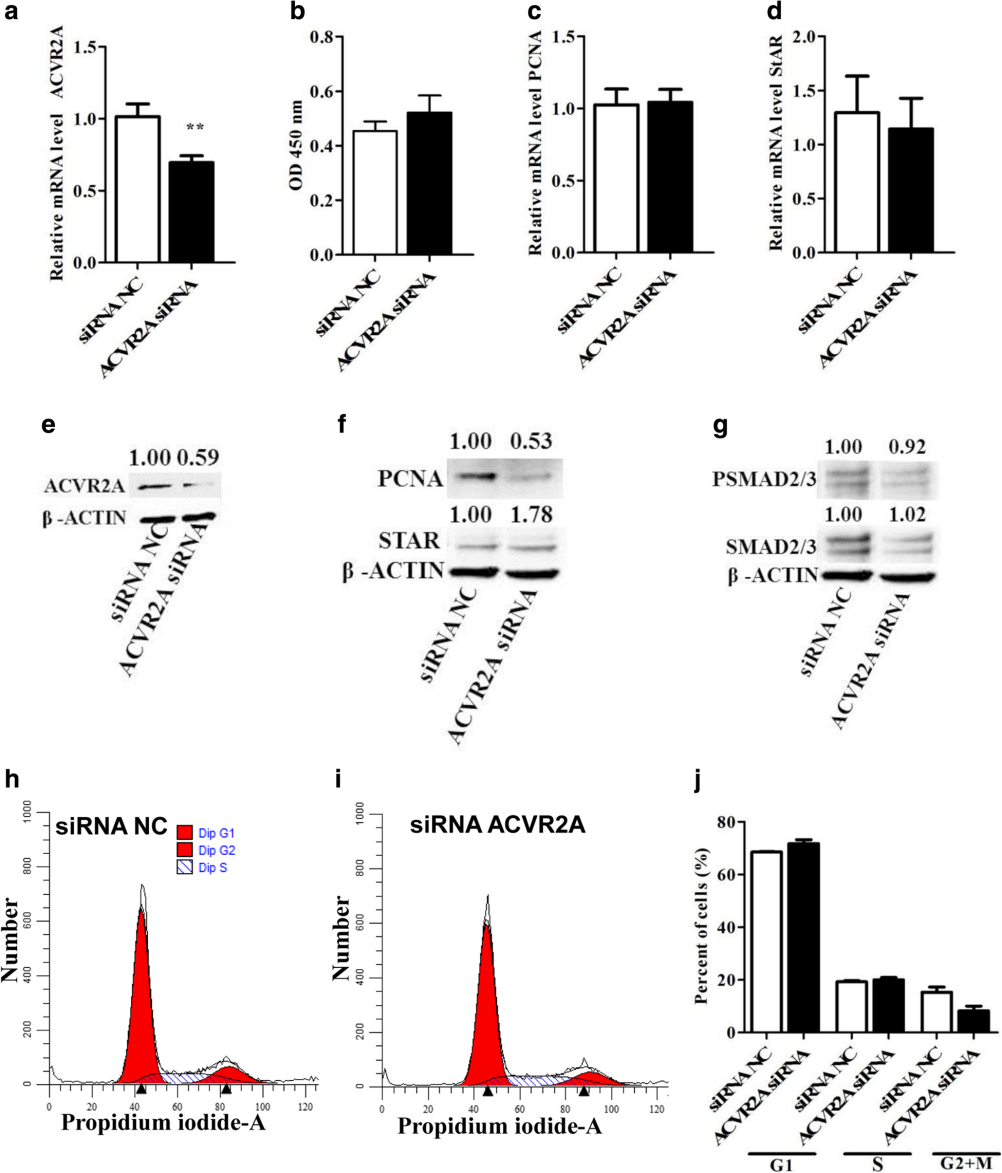
Effect of the sequence-specific knockdown of ACVR2A in bovine granulosa cells. The mRNA (**a**) and protein level (**e**) of ACVR2A in bovine granulosa cells transfected with ACVR2A siRNA or siRNA negative control (NC). The proliferation activity in granulosa cells transfected with ACVR2A siRNA or siRNA negative control (NC) (**b**). The mRNA (**c**) and protein level (**f**) of PCNA, and the mRNA (**d**) and protein level of (**f**) of STAR in granulosa cells transfected with ACVR2A siRNA or siRNA negative control (NC). The protein expression level of SMAD2/3 and PSMAD2/3 in granulosa cells transfected with siRNA NC or ACVR2A siRNA (**g**). Flow cytometric analysis showing the cell cycle distribution (G1/G0, S and G2/M phases) of propidium iodide (PI)-labeled bovine granulosa cells transfected with ACVR2A siRNA (**i**) or siRNA negative control (NC) (**h**). Bar graphs showing the percentages of cells in G1/G0, S and G2/M phases in granulosa cells transfected with ACVR2A siRNA or siRNA negative control (NC) (**j**). The bar graphs indicate the mean ± SEM (**p < 0.01). Numbers above western blots represent relative protein density

Similarly, to investigate the possible involvement of *ACVR2A* in bGCs proliferation and differentiation and to further validate the regulatory role of the miR-424/503 cluster in *ACVR2A* expression, we suppressed *ACVR2A* using small interfering RNA (siRNA). Bovine GCs transfected with the ACVR2A siRNA showed a significant reduction in the expression of both *ACVR2A* mRNA (*P* < 0.01) and protein compared to cells transfected with the siRNA negative control (Fig. 7a, e). The transfection of bGCs with the ACVR2A siRNA did not result in any measurable effect on the proliferation of bGCs compared to control siRNA-transfected bGCs (Fig. 7b). Similarly, there was no noteworthy change in the expression of the marker of proliferation *PCNA* following the suppression of the *ACVR2A* gene (Fig. 7c). However, western blot analysis showed reduced expression of the PCNA protein (Fig. 7f). Flow cytometric analysis showed no change in G1/S cell cycle transition (Fig. 7j). Further, the suppression of the *ACVR2A* gene using siRNA had no effect on the expression of the STAR gene (Fig. 7d). Western blot analysis showed that the expression of SMAD2/3 and phosphorylated SMAD2/3 was slightly decreased in siRNA-transfected cells compared to cells transfected with the negative control (7G).

### Activin treatment decreased *SMAD7* and miRNA-424/503 cluster members expression in bovine granulosa cells through activin signalling pathway

**Fig. 8:**
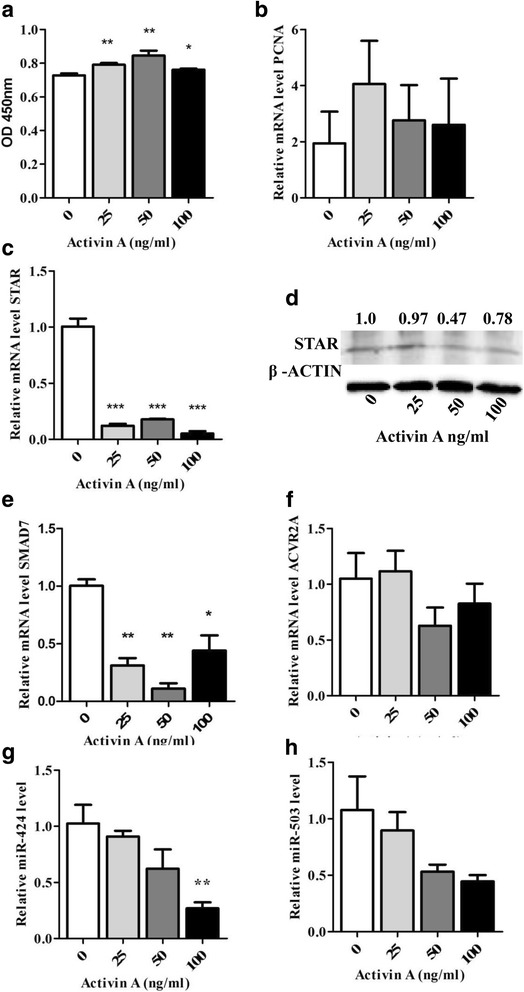
Dose-dependent activin A treatment increased bovine granulosa cell proliferation, reduced the SMAD7 and miR-424/503 expression levels. Cell proliferation assay (**a**) after dose-dependent Activin A treatment (Activin A: 25 ng/mL, 50 ng/mL and 100 ng/mL). The relative expression levels of PCNA (**b**), STAR (**c**), SMAD7 (**e**), ACVR2A (**f**), miR-424 (**g**) and miR-503 (**h**) in granulosa cells treated Activin A. The protein expression of STAR in granulosa cells treated with treated Activin A (**d**). The bar graphs indicate the mean ± SEM (**p < 0.01, ***p < 0.001). Numbers above western blots represent relative protein density

Activin has been implicated in various intra-ovarian roles, including granulosa cell proliferation, follicular luteinization retardation and the recruitment of R-SMADs to activate the activin signalling pathway. Here, we investigated the effect of Activin A treatment on the proliferation of granulosa cells and on miR-424/503 cluster expression. We treated bGCs with different doses of Activin A (25 ng/mL, 50 ng/mL and 100 ng/mL). We found that Activin A treatment increased proliferation of bovine granulosa cells in dose-dependent manner (Fig. 8a). Activin A treatment significantly decreased (*P* < 0.001) the *STAR* expression compared to control cells (Fig. 8c), which indicates a decrease in bGCs differentiation. Further, we also noticed that Activin A treatment in dose dependent manner significantly decreased the expression of *SMAD7* (*P* < 0.01; Fig. 8e); however, there was no change in the expression of *ACVR2A* (Fig. 8f). Interestingly, we observed a dose-dependent reduction in miR-424/503 expression upon treatment with Activin A. miRNA-424 was significantly decreased (*P* < 0.05; Fig. 8g) at the 100 ng/mL dose of Activin A. This result indicates that there might be negative feedback loop between Activin A and the miR-424/503 cluster. These results indicated that there could be possibility that miR-424/503 might be involved in fine tuning of the activin signalling pathway.

## Discussion

Folliculogenesis is a highly dynamic and well-coordinated process in which granulosa cells support follicular growth and development by producing hormones, autocrine and growth factors, and cytokines [5, 6] under the control of several molecular mechanisms and pathways. Increasing evidence supports the idea that miRNAs are one of the molecular mechanisms that epigenetically regulate the follicular development process [12, 13, 23, 40]. Several studies have shown that miRNAs regulate cumulus cell development and granulosa cell function by targeting TGF-β superfamily members [41–44]. In our previous study, we demonstrated that the miR-424/503 cluster was upregulated in bovine granulosa cells from preovulatory dominant follicles compared to those derived from subordinate follicle [23], which indicated the potential involvement of the miR-424/503 cluster in granulosa cell function to support ovulation. In the present study, we have demonstrated the role of miR-424/503 in bovine granulosa cell function through targeting *SMAD7* and further through coordinating activin signalling pathway. *SMAD7* and *ACVR2A* genes, which are important in reproduction [36, 37, 45], were identified as a putative targets of the miR-424/503 cluster, and subsequently validated by luciferase assay (Fig. 2). Further, overexpression of the miR-424/503 cluster reduced the expression of *SMAD7* and *ACVR2A* mRNA levels. These results suggested that the miR-424/503 cluster members target *SMAD7* and *ACVR2A* genes. However, since one miRNA could target some other potential target genes, we also believed that in addition to *SMAD7* and *ACVR2A genes* several other target genes of these miRNAs may also be involved in regulating bovine granulosa cell functions.

The present study demonstrates that granulosa cell proliferation was significantly increased upon the overexpression of miR-424, which was accompanied by upregulation of the *PCNA* gene. The evidence of the effect of modulating the expression of the miR-424/503 cluster members on granulosa cell proliferation was accompanied by a shift in the proportion of cells from the G0/G1-phase to the S-phase of the cell cycle. This finding is consistent with those of several reports demonstrating that miRNAs are essentially involved in the regulation of granulosa cell proliferation [13, 24, 46–48], which is necessary for follicular growth and creation of the unique micro-environment for oocyte maturation [49]. Furthermore, the proliferating granulosa cells in growing follicles depend on growth factors for their survival, which promote the G1-. to S-phase transition of the cell cycle and prevent apoptosis in granulosa cells with a low-progesterone environment that helps to persist and sustain the dominant follicle [50–52]. Interestingly, the present study showed a significant effect of miR-424 on granulosa cell proliferation compared to miR-503. This differential role of miR-424 could be associated with the fact that relative abundance of miR-424 was found to be high compared to miR-503 in granulosa cells of preovulatory dominant follicles in our previous study [23].

Several studies have shown that TGF-β superfamily members involved in granulosa cell proliferation are post-transcriptionally regulated by several classes of miRNAs [41, 47, 53]. Further, studies suggested *SMAD7* antagonizes TGF-β superfamily, and acts as potential regulator of ovarian function [36, 45]. In the present study, we demonstrated that *SMAD7*, which belongs to the TGF-β signalling pathway, is a direct target of miR-424/503 cluster members. Further, we have also shown knocking down of inhibitor SMAD (SMAD7) enhanced granulosa cell proliferation in in vitro, which was accompanied by increased expression of proliferation marker gene PCNA and further substantiated by granulosa cell cycle transition from G1- to S-phase. However, the function of SMAD7 in the ovary remains poorly understood. In one of the study, [45]) showed that SMAD7 expresses in granulosa cells and subject to regulation by intraovarian growth factors from the TGF-β superfamily. In the same study small interfering RNA demonstrated that SMAD7 acted a negative regulator of TGFβ1 and revealed a link between SMAD7 and GDF9-mediated oocyte paracrine signalling, an essential component of oocyte–granulosa cell communication. This suggested that SMAD7 function during follicular development via preferentially antagonizing and/or fine-tuning TGF-β superfamily signalling involved in the regulation of granulosa cell function [45]. Further, in another study [42], it was reported that MiR-92a inhibits porcine ovarian granulosa cell apoptosis by targeting Smad7 gene. In our study, we also noticed the similar finding that miR-424 promotes bovine granulosa cell proliferation by targeting the SMAD7 gene.

This suggests that miR-424/503 cluster member might be involved to control the activin signalling pathway through regulation of the inhibitory effect of *SMAD7*. Similarly, the cell cycle analysis results showed that overexpression of miR-424/503 resulted significant number of cells to be at S-phase compared to the untreated controls, which further validated proliferation assay of bovine granulosa cells. However, inhibition of both miRNAs did not result any change in cell cycle status of the granulosa cells compared to the untreated controls.

Addition of Activin A in in vitro cultured granulosa cells enhanced cell proliferation in dose-dependent manner (Fig. 8a), as it has been reported previously [41, 54]. Similarly, treatment of granulosa cells with varying doses (25, 50, 100 ng/mL) of Activin A sharply decreased the mRNA and protein expression of *STAR* (Fig. 8d). Similar studies also have shown that Activin A downregulates *STAR* expression and progesterone production in granulosa cells through SMAD2/3 phosphorylation [55], which ultimately helps to prevent or delay the premature luteinization of bovine granulosa cells [56]. We showed that similar to Activin A, the overexpression of the miR-424/503 cluster members clearly reduced *STAR* expression, which suggests the potential role of the miR-424/503 cluster members in the establishment and maintenance of the dominant follicle and further indicate involvement of miR-424/503 cluster members in activin signalling pathway.

**Fig. 9:**
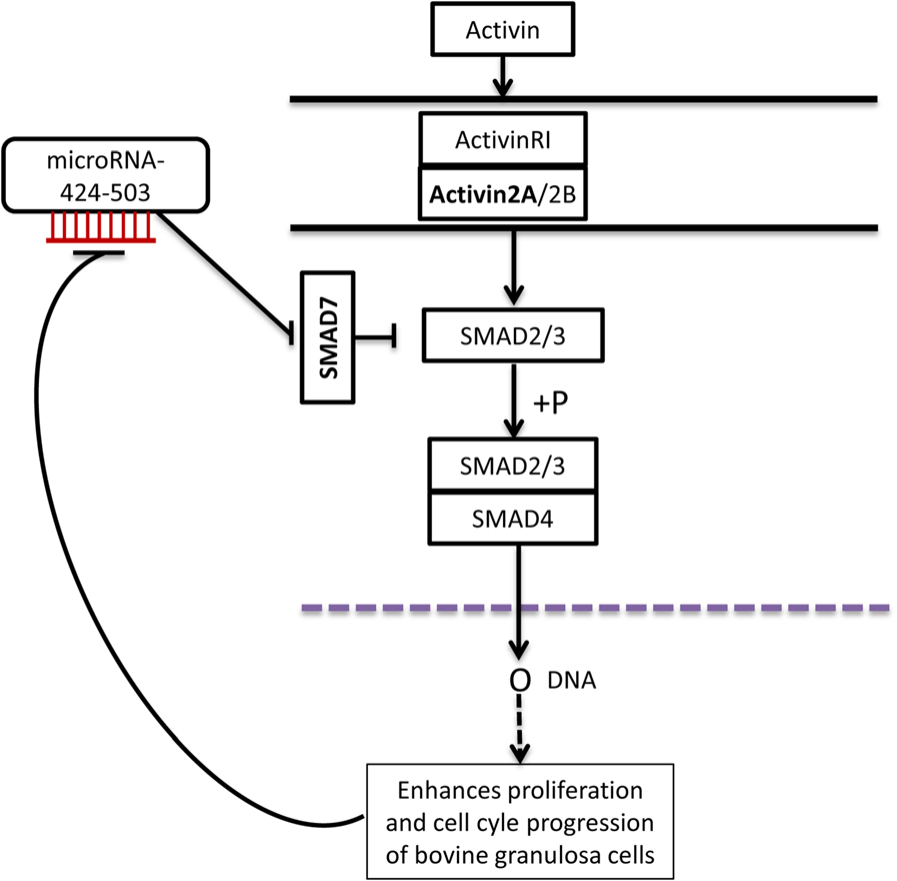
Proposed hypothetical model depicting the involvement of miR-424/503 cluster in the Activin signalling pathway by targeting SMAD7. Increased expression of miR-424/503 cluster suppresses the expression of SMAD7 which eventually increases the phosphorylation of SMAD2/3 and subsequently promotes the granulosa cell proliferation and cell cycle progression. Thus, when granulosa cells are treated with Activin A, the SMAD7 expression is suppressed, but the ACVR2A expression was maintained and leading to enhanced cell proliferation. At the time when the cells reach peak proliferation activity, the miR-424/503 expression was suppressed indicating the presence of negative feed back loop between the expression of miR-424/503 and cell proliferation activity in Activin A treated cells

The TGF-β - SMAD pathways are known to be an integral part of a range of biological processes and are errantly activated or inactivated under various biological conditions [57, 58]. SMAD proteins are potentially involved in controlling the transcription of a variety of miRNA genes, and this transcriptional activation of miRNAs has distinct physiological significance [58]. For instance, TGF-β induces both miR-216a and miR-217 in glomerular mesangial cells via SMAD binding elements (SBEs) in the miR-216 promoter [59]. In contrast, TGF-β-induced SMAD3/4 complex binding to the miR-24 promoter inhibits the expression of miR-24 in myoblasts [60]. Interestingly, dose-dependent activin A treatment reduced the expression of miR-424/503, whereas at a higher dose (100 ng/ml) significantly reduced the expression of miR-424 (Fig. 8g). This indicates possibility of negative feedback loop between the miR-424/503 cluster members and activin-SMAD2/3 signalling. Here, we proposed a hypothetical model illustrating the involvement of the miR-424/503 cluster members in regulating the activin signalling pathway (Fig. 9). In line to this, a report by Wang et al [53], which showed the presence of a potential feedback loop between miRNAs and the target gene.

Follicular growth and development is a dynamic and complex but nonetheless tightly harmonized process where activin plays an important role by enhancing the proliferation and reducing the differentiation of granulosa cells [61–63]. Several studies have shown that the dysfunction of granulosa cells may contribute to the abnormal folliculogenesis observed in ovarian pathophysiology [64–66], although the underlying mechanism remains to be determined. Furthermore, it is also important that granulosa cells should proliferate in controlled manner to avoid granulosa cell tumours, which account for 2–3% of ovarian malignancies [67]. Here, we reveal for the first time that the miR-424/503 cluster members are involved not only in the proliferation of granulosa cells but also in the balancing of the activin signalling pathway through regulating the expression of *SMAD7* and *ACVR2A* in bovine granulosa cells. This coordination of the activin signalling pathway through miRNAs might lead to healthy granulosa cells by avoiding any deviation from normalcy, like granulosa cell tumours. Previous studies have shown that miRNA-424-5p suppresses the expression of *SOCS6* in pancreatic cancer [68] and inhibits the Akt3/E2F3 axis and tumour growth in hepatocellular carcinoma [30]. Here, we suggest that perturbations in the expression of the miRNA-424/503 cluster members might result in ovarian disorders such as PCOS (polycystic ovarian syndrome) and granulosa cell tumours. Therefore, these results suggest that an optimal miRNA milieu is required for normal cellular and tissue function. The present study and other related miRNA studies provide insights into the molecular mechanisms underlying the regulation of granulosa cell functions and can facilitate a better understanding of follicular development and the pathophysiology of some reproductive disorders for the improvement of fertility treatments.

## Conclusion

In the present study, the luciferase assay showed *SMAD7* and *ACVR2A* genes to be the target genes of the miRNA-424/503 cluster members. Overexpression of miR-424/503 cluster members promoted granulosa cell proliferation and cell cycle progression accompanied by downregulation of the *SMAD7* and *ACVR2A* mRNA expression level. On the other hand, knockdown of the *SMAD7* not the *ACVR2A* mRNA expression level in granulosa cells resulted in similar phenotype characteristics that were obtained by overexpression of miR-424/503 cluster. Thus, the interplay between the miR-424/503 cluster members and *SMAD7* gene might be involved in the complex and coordinated process of bovine granulosa development. Nevertheless, apart from *SMAD7* gene, other target genes of the miRNA-424/503 cluster members might be involved in the regulation of bovine granulosa cell proliferation and cell cycle progression. Thus, additional study is required to further screen and identify downstream genes of miR-424/503 cluster members that are associated with granulosa cell function.

## Additional file

Additional file 1: **Table S1.** Sequence specific primers used for pmirGLO and 3´-UTR amplification of the SMAD7 and ACVR2A gene harboring binding site for miR-424-5p and miR-503-5p. **Table S2.** Sequence specific primers used for analysis of the relative expression of genes. (PDF 83 kb)

## Abbreviations

ACVR2A: Activin receptor type 2 A
bGCs: Bovine granulosa cells
cDNA: Complementary DNA
DMEM: Dulbecco modified eagle medium
FBS: Fetal bovine serum
miRNA-424/503 cluster: microRNA-424/503 cluster
NC: Negative controls
PCNA: Proliferating cell nuclear antigen
qRT-PCR: Quantitative real time polymerase chain reaction
RBC: Red blood cell
SiRNA: Small interfering RNA
SMAD2: SMAD family member 2
SMAD3: SMAD3 family member 3
SMAD7: SMAD7 family member 7
STAR: Steroidogenic acute regulatory protein
