# Supplementary material for: MicroRNA-424/503 cluster members regulate bovine granulosa cell proliferation and cell cycle progression by targeting SMAD7 gene through activin signalling pathway
Source: J Ovarian Res. 2018 May 1;11:34. doi: 10.1186/s13048-018-0410-3 (PMC5930509; doi:10.1186/s13048-018-0410-3)
Supplement: Supplementary file 1 — Table S1. Sequence specific primers used for pmirGLO and 3´-UTR amplification of the SMAD7 and ACVR2A gene harboring binding site for miR-424-5p and miR-503-5p. Table S2. Sequence specific primers used for analysis of the relative expression of genes. (PDF 83 kb) [file 13048_2018_410_MOESM1_ESM.pdf]

Table S1. Sequence specific primers used for pmirGLO and 3´-UTR amplification of the SMAD7 and ACVR2A gene harboring binding site for miR-424-5p and miR-503-5p

**Accession number**	**Genes**	**Primer sequence**	**Size (bp)**
FJ376737	pmirGLO	F: 5´-GCAAGATCGCCGTGTAATTC-3´<br>R: 5´-CTTTCGGGCTTTGTTAGCAG-3´	107
XM_005224232.3	SMAD7-miR/424-503	F: 5´-GCAT<u>GAGCTC</u>AGCAGGCCACACTTCAAACT<br>R: 5´-ATG**CTCGAG**GGACGAGAAGAAGAAAACCAACC	138
NM_174227	ACVR2A-miR-424	F: 5´-GC<u>GAGCTC</u>TTGCATTTGCTGTTGTGTTTCT-3´<br>R: 5´-TAT**CTCGAG**TAGCAACCGTGGAACTGAGG-3´	201
NM_174227	ACVR2A-miR-503	F: 5´-TA<u>GAGCTC</u>ACACACTGAGAAACAGGACTCT-3´<br>R: 5´-TAT**CTCGAG**CCTTGATTTGGAGAGGGCCA-3´	217

Underlined: SacI recognition site (GAGCT|C). Bold: XhoI recognition site (C|TCGAG)

Table S2. Sequence specific primers used for analysis of the relative expression of genes

Accession number	Genes	Primer sequence	Product size (bp)
NM_173979	β-ACTIN	F: 5´-TGTCCACCTTCCAGCAGAT-3´<br>R: 5´-TCACCTTCACCGTTCCAGT-3´	249
NM_001034494	PCNA	F: 5´-CACCAGCATGTCCAAAATAC-3´<br>R: 5´-CTGAGATCTCGGCATATACG-3´	192
NM_174189	STAR	F: 5´-AAATCCCTTTCCAAGGTCTG-3´<br>R: 5´-ACCAGCATTTCTGCTACTGC-3´	204
NM_001192865.1	SMAD7	F: 5´-GTGGCATACTGGGAGGAGAA-3´<br>R: 5´-TTGTTGTCCGAATTGAGCTG-3´	128
NM_174227	ACVR2A	F: 5´- CAGAGAAACGAGGCACCAGT-3´<br>R: 5´- GGCCATCTTTTAGGCCAGGT-3´	186
